# Actin cytoskeleton differently regulates cell surface organization of GPI-anchored proteins in polarized epithelial cells and fibroblasts

**DOI:** 10.3389/fmolb.2024.1360142

**Published:** 2024-05-07

**Authors:** Stéphanie Lebreton, Simona Paladino, Mickaël Lelek, Marc Tramier, Christophe Zimmer, Chiara Zurzolo

**Affiliations:** ^1^ Institut Pasteur, Unité de Trafic Membranaire et Pathogenèse, Paris, France; ^2^ Department of Molecular Medicine and Medical Biotechnology, University of Naples Federico II, Naples, Italy; ^3^ Imaging and Modeling Unit, Department of Computational Biology, Institut Pasteur, Paris, France; ^4^ Université Rennes, Centre National de la recherche scientifique, IGDR (Genetics and Development Institute of Rennes), Unité mixte de receherche 6290, Rennes, France; ^5^ Rudolf Virchow Center, University of Würzburg, Würzburg, Germany

**Keywords:** GPI-anchored proteins, actin cytoskeleton, polarized epithelial cells, surface organization, protein clustering, imaging techniques, protein sorting and trafficking

## Abstract

The spatiotemporal compartmentalization of membrane-associated glycosylphosphatidylinositol-anchored proteins (GPI-APs) on the cell surface regulates their biological activities. These GPI-APs occupy distinct cellular functions such as enzymes, receptors, and adhesion molecules, and they are implicated in several vital cellular processes. Thus, unraveling the mechanisms and regulators of their membrane organization is essential. In polarized epithelial cells, GPI-APs are enriched at the apical surface, where they form small cholesterol-independent homoclusters and larger heteroclusters accommodating multiple GPI-AP species, all confined within areas of approximately 65–70 nm in diameter. Notably, GPI-AP homoclustering occurs in the Golgi apparatus through a cholesterol- and calcium-dependent mechanism that drives their apical sorting. Despite the critical role of Golgi GPI-AP clustering in their cell surface organization and the importance of cholesterol in heterocluster formation, the regulatory mechanisms governing GPI-AP surface organization, particularly in the context of epithelial polarity, remain elusive. Given that the actin cytoskeleton undergoes substantial remodeling during polarity establishment, this study explores whether the actin cytoskeleton regulates the spatiotemporal apical organization of GPI-APs in MDCK cells. Utilizing various imaging techniques (number and brightness, FRET/FLIM, and dSTORM coupled to pair correlation analysis), we demonstrate that the apical organization of GPI-APs, at different scales, does not rely on the actin cytoskeleton, unlike in fibroblastic cells. Interestingly, calcium chelation disrupts the organization of GPI-APs at the apical surface by impairing Golgi GPI-AP clustering, emphasizing the existence of an interplay among Golgi clustering, apical sorting, and surface organization in epithelial cells. In summary, our findings unveil distinct mechanisms regulating the organization of GPI-APs in cell types of different origins, plausibly allowing them to adapt to different external signals and different cellular environments in order to achieve specialized functions.

## Introduction

Glycosylphosphatidylinositol (GPI)-anchored proteins, attached to the external leaflet of the plasma membrane via their glycolipid anchor, represent 0.5% of total proteins in eukaryotes. In mammals, more than 150 GPI-APs have been identified, and they play diverse functions, ranging from enzymatic activity, cell adhesion, signaling, neuritogenesis, and immune response (Lebreton et al., 2018; Müller and Müller, 2023).

In polarized epithelial cells, GPI-APs are mostly enriched at the apical domain of the plasma membrane where they exert their functions (Lebreton et al., 2018; Lebreton et al., 2019). As with all plasma membrane proteins and perhaps even more so, given their unique glycolipid anchoring characteristics, the biological activities of GPI-APs are governed by their spatiotemporal organization within the membrane.

At the apical surface of MDCK cells, approximately 30%–35% of GPI-APs is organized in cholesterol-independent homoclusters of 3–4 molecules (accommodating a single GPI-AP species) that are required for cholesterol-dependent heterocluster formation (accommodating different GPI-AP species) (Paladino et al., 2014). Furthermore, by using direct stochastic optical reconstruction microscopy coupled to pair correlation analysis (pc-STORM) (Sengupta et al., 2011; Lelek et al., 2021), it has been shown that GPI-APs at the apical membrane are non-randomly distributed but instead confined in cholesterol-independent domains of an average diameter of ∼ 67 nm (Paladino et al., 2017). This complex organization has a fundamental functional relevance (Lebreton et al., 2018). For instance, the catalytic activity of the GPI-AP placental alkaline phosphatase (PLAP) is strictly dependent on this fine organization (Paladino et al., 2014). Similarly, the existence of the urokinase-type plasminogen activator (uPA) receptor (uPAR) as monomer, dimer, and higher aggregates at the cell surface of epithelial HEK293 cells modulates its differential ligand binding, thus activating distinct intracellular pathways (Cunningham et al., 2003; Caiolfa et al., 2007; Hellriegel et al., 2011). Intriguingly, the GPI-AP folate receptor is internalized via a clathrin and dynamin-independent pathway with respect to its transmembrane counterpart, resulting in a diverse cellular fate that directly impacts on the folate uptake (Mayor et al., 2014).

In polarized epithelial cells, apical plasma membrane organization and biological activities of GPI-APs are strictly dependent on their Golgi clustering that also governs their apical sorting (Lebreton et al., 2008; Paladino et al., 2008; Paladino et al., 2014; Lebreton et al., 2019; Lebreton et al., 2021). Several studies uncovered that cholesterol and calcium levels are master regulators of Golgi GPI-AP clustering (Lebreton et al., 2008; Paladino et al., 2008; Paladino et al., 2014; Lebreton et al., 2019; Lebreton et al., 2021).

However, besides the role of Golgi GPI-AP clustering in their apical surface organization and of cholesterol in heterocluster formation, how apical membrane GPI-AP organization is regulated in epithelial cells in the context of polarity remains elusive.

At the cell surface of fibroblasts, GPI-APs are organized in cholesterol- and actin-dependent nanocluster accommodating different GPI-APs (Varma and Mayor, 1998; Sharma et al., 2004; Goswami et al., 2008), thus supporting an interplay between actin and cholesterol (Goswami et al., 2008). Of interest, the actin cytoskeleton undergoes substantial remodeling during polarity establishment (Yonemura et al., 1995; Li and Gundersen, 2008), prompting us to investigate whether the actin cytoskeleton regulates the spatiotemporal organization of GPI-APs at the apical surface of polarized MDCK cells.

By combining number and brightness (N&B) (Digman et al., 2008), fluorescence lifetime imaging microscopy (FLIM) (Padilla-Parra et al., 2008; Padilla-Parra et al., 2009), and pair correlation analysis of single-molecule localization microscopy (pc-STORM) (Rust et al., 2006; Sengupta et al., 2011), we report that actin perturbation does not compromise the organization of apical GPI-APs, including both homoclusters and heteroclusters, as well as their spatial confinement opposite to CHO cells. Therefore, our results support the interplay between cholesterol and actin in CHO cells but not in polarized MDCK cells.

Furthermore, we show that calcium chelation disrupts the organization of apical GPI-APs by impeding Golgi GPI-AP clustering, emphasizing the interplay among Golgi clustering, apical sorting, and surface organization in epithelial cells.

In conclusion, our data reinforce the existence of distinct mechanisms governing the organization of GPI-APs in polarized epithelial and fibroblastic cells. Specifically, in fibroblasts GPI-AP organization relies on cholesterol and actin at the cell surface, while in epithelial cells cholesterol- and calcium-dependent Golgi clustering plays a direct role in governing GPI-AP organization at the apical membrane but actin does not. This different organization may be raised to the different embryonic origin of these cells and could ensure that epithelial and fibroblastic cells are capable in adapting to different cellular environments and stimuli in order to achieve the specialized functions unique to each cell type.

## Results and discussion

### Actin cytoskeleton differently regulates cell surface GPI-AP homo- and confined clusters in fibroblastic and polarized MDCK cells

**FIGURE 1:**
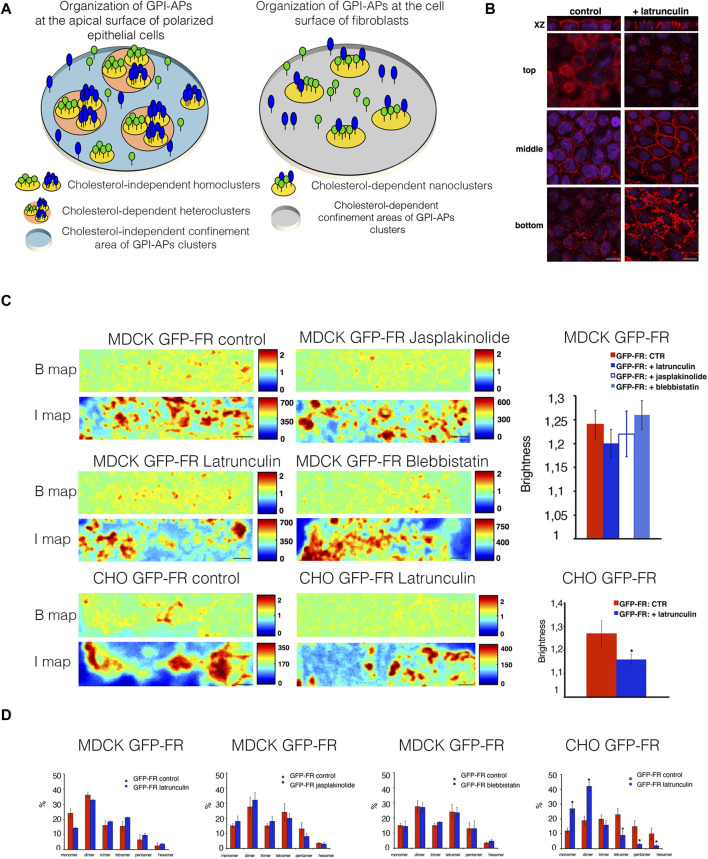
Alteration of actin dynamics does not affect the apical homocluster organization of GPI-APs in MDCK cells. **(A)** Model of GPI-AP organization at the apical membrane of polarized epithelial cells (on the left) and at the cell surface of fibroblasts (on the right). **(B)** Polarized MDCK cells grown on filters were incubated in the presence of 6 μM latrunculin A for 5 min, fixed, and stained with rhodamine-conjugated phalloidin to reveal the actin cytoskeleton. Nuclei were stained with DAPI. Serial confocal sections were collected using a laser-scanning microscope (LSM 510 META, Carl Zeiss Microimaging Inc.) equipped with a plan apo ×63 oil-immersion (NA 1.4) objective lens. Top, middle, and bottom correspond to Z-slices taken at 1–1.5 μm, 4–5 μm, and 9–10 μm of the cell monolayer, respectively. Scale bars, 9 μm. **(C)** Polarized MDCK cells expressing GFP-FR were treated or not treated with latrunculin, jasplakinolide, and blebbistatin. CHO cells expressing GFP-FR after 2 days in culture were treated or not with latrunculin. Then, these cells were imaged *in vivo* for N&B. Brightness (B) and fluorescence intensity (I) maps of an area imaged by N&B (scanning ROIs of 256 × 64 pixels over time) of the representative cell in control conditions or upon actin perturbations are shown. Scale bars, 0.9 μm. Quantification of the brightness of GFP-FR from three independent experiments is plotted, *n* > 50 cells. Error bars, ± SD. *p < 0.0003, Student’s *t-*test. **(D)** Graphical representation of the percentage of pixels falling into the different classes of B-values (from monomer to hexamer) on the basis of the calibration curve [see Methods and Paladino et al. (2014)]. Values are expressed as the mean of three independent experiments, *n* > 25 cells. Error bars, ± SD. *p < 0.0001, Student’s *t-*test.

Previous studies showed that at the cell surface of fibroblastic cells, GPI-APs are organized in cholesterol-dependent nanoclusters that are confined in cholesterol-dependent domains of 65–70 nm in diameter (Sharma et al., 2004; Goswami et al., 2008; Sengupta et al., 2011; Paladino et al., 2017). Differently, at the apical surface of polarized epithelial cells, GPI-APs are organized in cholesterol-independent homoclusters that can further coalesce into cholesterol-dependent heteroclusters; both are confined to cholesterol-independent areas of ∼65–70 nm in diameter (Paladino et al., 2014; Paladino et al., 2017), therefore highlighting a different cholesterol dependency in GPI-AP organization at the cell surface of both cell types (Figure 1A).

To analyze the role of actin cytoskeleton dynamics in GPI-AP organization, fully polarized MDCK cells, grown for 3 days on polycarbonate filters, were incubated with latrunculin A (6 μM for 5 min), a compound known to destabilize the actin cytoskeleton by promoting actin depolymerization in different cell types including polarized MDCK cells (Figure 1B) (Bubb et al., 1994; Bubb et al., 2000; Morton et al., 2000). Figure 1B reveals that this short treatment is sufficient to alter the actin cytoskeleton underneath the apical plasma membrane (top section).

By applying N&B analysis (Digman et al., 2008; Paladino et al., 2014) (see Materials and Methods), we measured the brightness of a model GPI-AP, GFP-FR [in which GFP is fused to the GPI attachment signal of the apically sorted folate receptor (FR)] at the cell surface of CHO cells and at the apical surface of polarized MDCK cells in order to determine the aggregation state (and number of molecules). In untreated control cells, the brightness of GFP-FR is 1.21 at the apical surface of polarized MDCK cells and 1.3 at the cell surface of CHO cells (Figure 1C), which corresponds to protein clusters containing three to four molecules, as previously shown (Paladino et al., 2014). Upon latrunculin A treatment, at the apical surface of polarized MDCK cells, the brightness values of GFP-FR are comparable to control conditions (1.21 and 1.23, respectively; Figure 1C), while in CHO cells, the brightness of GFP-FR decreases from 1.3 to 1.15, corresponding to GFP-FR monomer/dimers. These data reveal that GPI-APs behave differently in the two cell types and that in polarized epithelial cells, GPI-AP homoclustering is independent of the actin cytoskeleton. Moreover, to evaluate the contribution of actin dynamics in apical GPI-AP homoclusters, we monitor the brightness of GFP-FR upon jasplakinolide (5 μM for 5 min) known to stabilize actin cytoskeleton and blebbistatin (50 μM for 90 min) that alters myosin IIa activity (Straight et al., 2003; Goswami et al., 2008). We found no difference in the brightness of GFP-FR upon these treatments with respective control conditions (Figures 1C, D), as evidenced by the percentage of pixels falling in different classes of B-values (from monomer to hexamer) determined by extrapolation of the standard curve obtained by plotting experimental B-values of monomeric, dimeric, and trimeric GFP (see Methods, and Paladino et al., 2014), further supporting that apical GPI-AP homoclusters are independent of the acto-myosin cytoskeleton. In order to confirm that the actin cytoskeleton does not regulate the clustering of GPI-APs at the apical surface of polarized MDCK cells, fully polarized MDCK cells grown on filters were treated for 30 min with latrunculin A at 25 μM, as previously shown (Goswami et al., 2008). This treatment affects the whole organization of the actin cytoskeleton of MDCK cells, perturbing the actin network both at the apical, lateral, and basal surface of the cells (Supplementary Figure S1A). Upon this treatment, comparable amount of GFP-FR was purified by velocity gradient in high-molecular weight complexes, as in control conditions (Supplementary Figure S1B), indicating that GPI-AP clusters are unaltered even upon severe actin perturbations. Together with previous findings showing that latrunculin A does not affect homoclustering of GFP-FR at the Golgi (Lebreton et al., 2021), our data strongly support that membrane organization of GPI-APs does not rely on the actin cytoskeleton.

**FIGURE 2:**
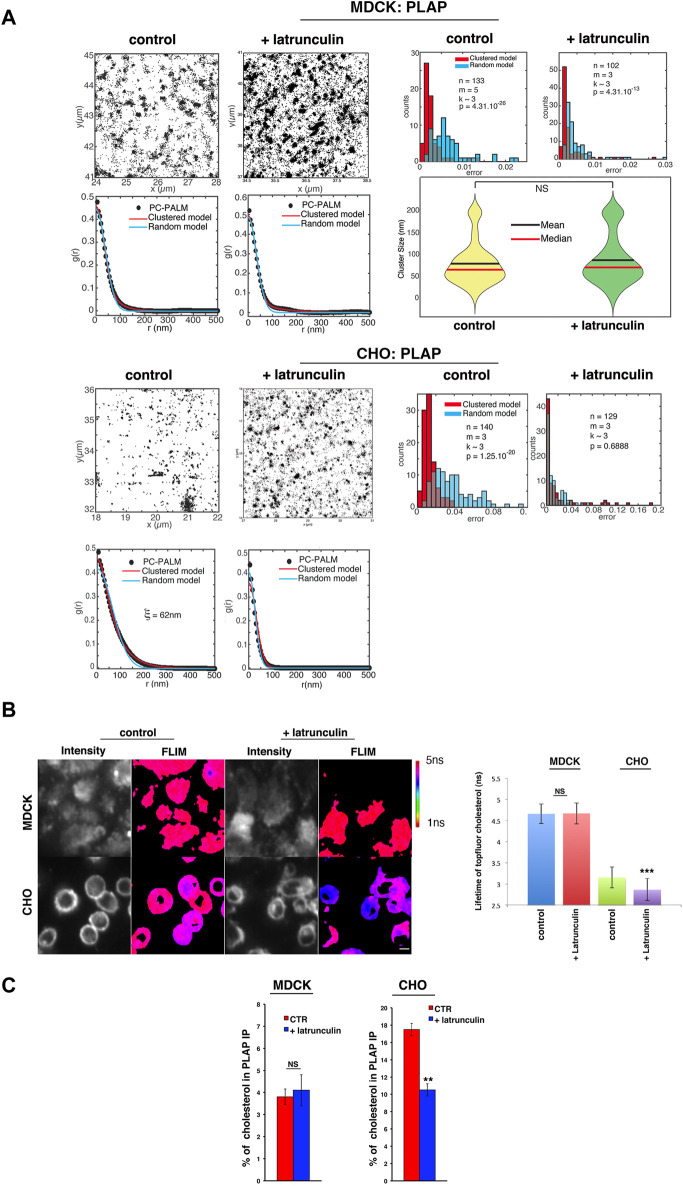
Alteration of actin dynamics does not affect the apical GPI-AP confined cluster in MDCK cells. **(A)** Polarized MDCK cells or CHO cells expressing PLAP were treated or not treated with 6 μM latrunculin A for 5 min, imaged using dSTORM, and subjected to pc-STORM analysis. Representative images (4 × 4 μm area) of STORM localizations of PLAP in control conditions and upon latrunculin treatment at the apical membrane of MDCK or CHO cells are shown. The density of localizations is equivalent in all conditions (1,300–5,000 localization/µm^2^). The pair correlation function and the distribution of mean squared errors between the data and the fitted model are shown. Statistical analyses were computed from n areas (pc-STORM curves), m experiments, and k cells (on average) per experiment. Overlaps between the two histograms appear in grey. The Kolmogorov–Smirnov test is used to compare the two error distributions and to assess if the clustered model provides a significantly better fit than the random model. If the difference is not significant (*p* > 0.05), the molecular distribution is categorized as random. In polarized MDCK cells, both with or without latrunculin treatment, the pair correlation data are significantly better fitted with a clustered model than a random model (*p* < 10^−26^ and *p* < 10^−13^, respectively), indicating a clustered organization of PLAP in both cases. Violin plots show the distribution of PLAP cluster sizes, as obtained from the clustered model fits (mean and median are indicated; right lower panel with NS: not significant). In untreated CHO cells, the pair correlation data are significantly better fitted with a clustered model than a random model (*p* < 10^−20^), suggesting a clustered organization as well. Upon latrunculin A treatment, the pair correlation function is equally well fit by a clustered or a random model (*p* = 0.69), indicating a random organization of PLAP. **(B)** Polarized MDCK cells grown on the filter for 4 days and CHO cells grown on bottom-glass dishes for 2 days were loaded with TopFluor cholesterol (see Methods) and then treated or not with latrunculin A for 5 min before measuring its lifetime. On the left, intensity and mean fluorescence lifetime maps of TopFluor cholesterol in control or upon latrunculin treatment are shown. The lifetime scale is from 1 to 5 ns. Scale bars, 18 μm. On the right, histograms of TopFluor cholesterol lifetime (ns) in control conditions (blue and green bars) or upon latrunculin treatment (red and purple) are shown. Experiments were performed three times; *n* > 35 cells. Error bars, ± SD. *p < 0.0001, Student’s t-test. **(C)** After 4 or 2 days in culture, MDCK and CHO cells stably expressing PLAP were incubated for 6 h with [^3^H]-cholesterol in the culture medium and then chased for 24 h in fresh culture medium. Control- and latrunculin A-treated cells were lysed in buffer containing 1% TX-100, and lysates were immunoprecipitated with a specific antibody against PLAP. The radioactivity associated with PLAP immunoprecipitates and with total lysates was determined by liquid scintillation counting. The percentage of cholesterol in PLAP immunoprecipitates with respect to the total cell cholesterol of three independent experiments is shown. Error bars, ±SD. **p < 0.01, Student’s *t-*test.

Next, we assessed whether actin perturbation affects the spatial distribution of GPI-APs in polarized MDCK and CHO cells by single-molecule localization dSTORM coupled to pair correlation analysis (pc-STORM). In agreement with previous findings (Sengupta et al., 2011; Paladino et al., 2017), we found that PLAP is confined to clusters with diameters of ∼ 65–70 nm in polarized MDCK cells and at the cell surface of CHO cells (Paladino et al., 2017). However, latrunculin A treatment led to a random organization of PLAP in CHO cells (Supplementary Figure S1C); in polarized MDCK cells, PLAP remained organized in clusters of 65–70 nm in diameter upon latrunculin A treatment (Wilcoxon test: *p* = 0.375; Figure 2A). Thus, these data strengthen the notion that the surface organization of GPI-APs differs between these two cell types.

### Actin perturbation affects cholesterol distribution in CHO cells but not in MDCK cells

Importantly, treatments modifying cholesterol levels have been reported to affect the actin cytoskeleton in CHO cells (Goswami et al., 2008), suggesting an interplay between actin and cholesterol regulations. In order to define whether cholesterol and actin could be linked at the apical surface of polarized MDCK cells, we analyzed the lifetime of a fluorescent cholesterol analog, BODIPY-cholesterol derivative (TopFluor cholesterol), shown to closely mimic cholesterol (Holtta-Vuori et al., 2008; Ariola et al., 2009; Wustner et al., 2011), in control conditions and upon latrunculin A treatment both in CHO and MDCK cell lines. At the apical surface of polarized MDCK cells, the lifetime of the TopFluor-cholesterol is 4.71 ns (±0.23) and remains unchanged upon 5 min latrunculin A treatment (4.66 ns ± 0.24) (Figure 2B), while in CHO cells, the lifetime of TopFluor-cholesterol is 3.13 ns (±0.45) and decreases significantly to 2.88 ns (±0.33, *p* < 0.0001) upon 5 min latrunculin A (Figure 2B). These data fully support a relationship between the organization of the actin cytoskeleton and cholesterol in CHO cells, as previously mentioned (Sun et al., 2007; Goswami et al., 2008; Gowrishankar et al., 2012; Chubinskiy-Nadezhdin et al., 2013; Raghupathy et al., 2015), but not in polarized MDCK cells. Additionally, the difference in the lifetime of fluorescent cholesterol in control conditions in the two cell types supports a difference in the membrane environment surrounding this analog.

To gain further insights on the effect of the actin perturbation on the cholesterol content in the vicinity of GPI-APs, we loaded the cells with ^3^H-cholesterol and measured the amount of cholesterol in the PLAP immunoprecipitates in control condition and upon actin perturbation [see Methods section (Tivodar et al., 2006)] in both CHO and MDCK cells (Figure 2C). Notably, in CHO cells, treatment with latrunculin A reduced the amount of cholesterol in PLAP immunoprecipitates (from 18% to 11% of total cholesterol), while it did not have any effect in MDCK cells (Figure 2C).

These data are consistent with the current hypothesis that in fibroblasts, cholesterol and actin are inter-regulated and with the findings that perturbation of one or the other affects the cell surface organization of GPI-APs (Goswami et al., 2008; Raghupathy et al., 2015). However, this does not appear to be the case in polarized epithelial cells, where other mechanisms regulating GPI-AP organization must be at play.

### Perturbation of the actin cytoskeleton affects GPI-AP heterocluster organization at the apical surface of CHO cells but not in MDCK cells

**FIGURE 3:**
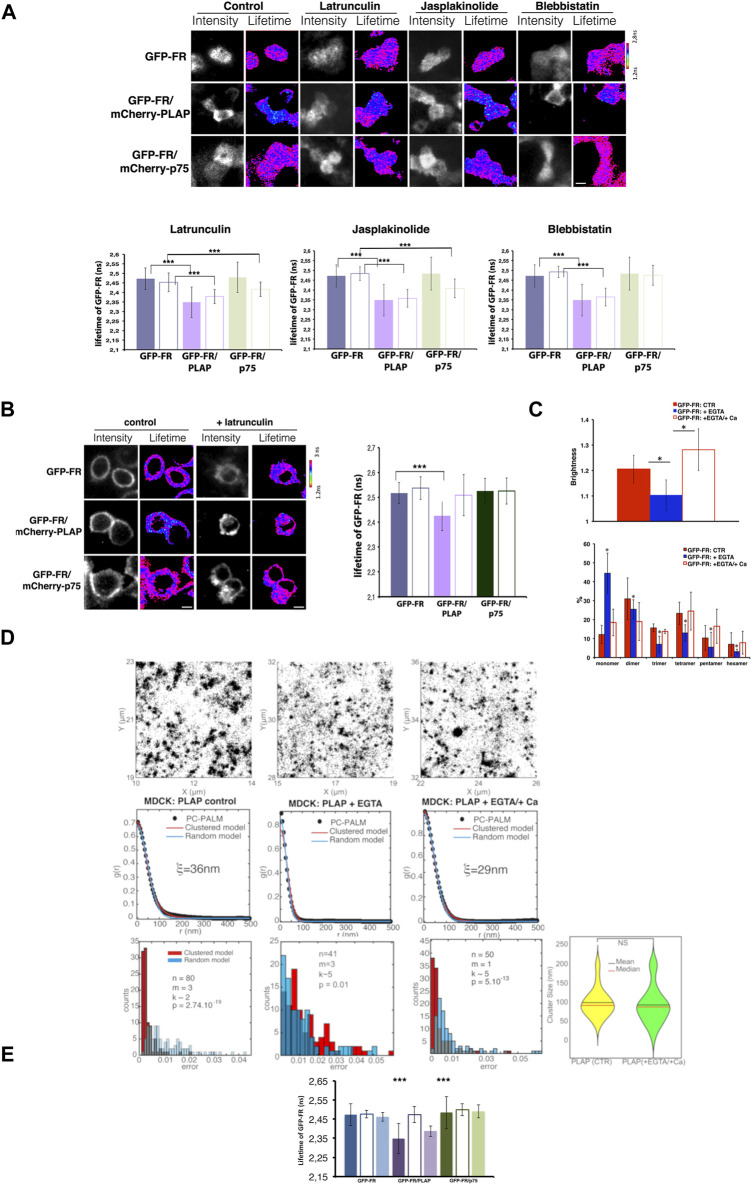
Alteration of actin dynamics does not affect the apical heterocluster organization of GPI-APs in MDCK cells, and EGTA chelation totally abolishes apical GPI-AP organization. **(A)** Intensity and mean fluorescence lifetime maps of GFP-FR alone or in the presence of either mCherry-PLAP (GFP-FR/mCherry-PLAP) or mCherry-p75 (GFP-FR/mCherry-p75) in control or upon the aforementioned treatments. Cells were imaged live by scanning ROIs of 140 × 140 pixels corresponding to four to six cells of the confluent polarized monolayer. Dark areas correspond to cells either not expressing the indicated protein or out of focus. The lifetime scale is from 1.2 to 2.8 ns. Scale bars, 9 μm. In the lower panels, histograms of GFP-FR lifetime (ns) alone (blue bars) or in combination with mCherry-PLAP (purple bar) or mCherry-p75 (green bars) in control conditions (colored bars) or upon latrunculin or jasplakinolide or blebbistatin treatment (pale colored bars) are shown. Experiments were performed three times; *n* > 35 cells. Error bars, ± SD. ***p < 0.0001 (comparing appropriate control with treated condition), Student’s *t-*test. It should be noted that as positive control, we used cholesterol depletion, which affected the FRET between GFP-FR and mCherry-PLAP and dispersed the heteroclusters [as previously shown in Figures 2C, D in Paladino et al. (2014)]. **(B)** Intensity and mean fluorescence lifetime maps of GFP-FR alone or in the presence of either mCherry-PLAP (GFP-FR/mCherry-PLAP) or mCherry-p75 (GFP-FR/mCherry-p75) in control or upon latrunculin A treatment. The lifetime scale is from 1.2 to 3 ns. Cells were imaged live by scanning ROIs of 140 × 140 pixels. Scale bars, 9 μm. Histograms of GFP-FR lifetime (ns) alone (blue bars) or in combination with mCherry-PLAP (purple bars) or mCherry-p75 (green bars) in control conditions (colored bars) or upon latrunculin A treatment (pale colored bars) are shown. Experiments were performed three times; *n* > 35 cells. Error bars, ±SD. ***p < 0.0001, Student’s t-test. **(C–E)** After 4 days in culture, polarized MDCK expressing GFP-FR were treated with EGTA, a chelator of calcium, followed or not by a replenishment of calcium for 3 h before imaging cells *in vivo* for N&B **(C)** or FLIM **(E)**; polarized MDCK cells expressing PLAP were subjected to the same treatment (EGTA alone or EGTA followed by calcium replenishment) before STORM analysis **(D)**. In **(C)**, on the top, quantification of the brightness of GFP-FR from three independent experiments either in control conditions (CTR, red bars) or upon calcium chelation (+EGTA, blue bars) or upon calcium replenishment (+Ca, empty red bars), *n* > 50 cells. Below, graphical representation of the percentage of pixels falling into different classes of B-values (from monomer to hexamer) on the basis of the calibration curve (Paladino et al., 2014) in different conditions. Values are expressed as the mean of three independent experiments, *n* > 30 cells. Error bars, ± SD. *p < 0.0001, Student’s *t-*test. In **(D)**, pair correlation analysis of dSTORM data on PLAP in control conditions and upon chelation of calcium (+EGTA) or replenishment (EGTA/+Ca) at the surface of polarized MDCK cells. In control cells, the pair correlation data are fitted significantly better with a clustered model than a random model (*p* < 10^−19^), revealing a clustered organization of PLAP. Upon chelation of calcium, the pair correlation data are fitted equally well with the random model and the clustered model (*p* > 0.1), suggesting a perturbation of the clustered organization of PLAP. In case of replenishment of calcium, the pair correlation data are statistically better fitted with a clustered model than a random model (*p* < 10^−13^), indicating reformation of clusters with a similar size of cluster compared to control condition (lower panel right). **(E)** Histograms of GFP-FR lifetime alone (blue bars) or in combination with mCherry-PLAP (purple bar) or mCherry-p75 (green bars) in control conditions (colored bars) or upon chelation of calcium (empty bars) or upon chelation and replenishment of calcium (pale colored bars). Experiments were performed three times, *n* > 35 cells. Error bars, ± SD. ***p < 0.001, Student’s *t-*test.

Next, by using FRET-FLIM, we analyzed whether the actin cytoskeleton was involved in regulating the relationship between two different GPI-APs (heteroclusters) (Padilla-Parra et al., 2008; Paladino et al., 2014). Specifically, we measured the lifetime of GFP-FR (donor) at the apical surface of polarized MDCK cells expressing GFP-FR alone, or in the presence of an acceptor (another GPI-AP, mCherry-PLAP, or a transmembrane apical protein mCherry-p75), upon latrunculin A, jasplakinolide, and blebbistatin treatments (Figure 3A). In all these conditions as in control cells, the lifetime of GFP-FR in the presence of mCherry-PLAP was statistically significantly decreased compared to the lifetime of GFP-FR alone (Figure 3A), indicating that the two proteins are still within FRET distance and that heteroclusters between GFP-FR and mCherry-PLAP are insensitive to actin perturbations. On the contrary, in CHO cells, the lifetime of GFP-FR, which decreases in the presence of mCherry-PLAP in control conditions, indicating that the two proteins are in close proximity (nanoclusters/heteroclusters), remained unchanged upon latrunculin A treatment, indicating that the two GPI-APs are not organized in heteroclusters/nanoclusters anymore (Figure 3B).

Interestingly, at the apical surface of polarized MDCK cells upon latrunculin A and jasplakinolide treatments, we detected a significant decrease in the lifetime of GFP-FR in the presence of the control transmembrane protein mCherry-p75 (from 2.48 ns ± 0.08 to 2.41 ns ±0.038 and 2.40 ns ±0.048, respectively, *p* < 0.0001) (Figure 3A), indicating the occurrence of FRET between these two proteins, which are not in close proximity in control conditions (Meder et al., 2006; Lebreton et al., 2008; Paladino et al., 2014). This latter result suggests that upon actin perturbation, the apical p75^NTR^ is no longer constrained by the actin meshwork and therefore comes in close proximity to GPI-APs, further reinforcing a role of the actin cytoskeleton in the spatiotemporal regulation of transmembrane proteins at the apical surface of polarized MDCK cells but not of the GPI-AP organization. Of note, in CHO cells, latrunculin A treatment did not lead to a decrease in GFP-FR lifetime in the presence of mcherry-p75, as in the case of polarized MDCK cells, further reinforcing differences in protein organization between polarized MDCK and CHO cells. Thus, taken together, these data demonstrate that GPI-APs have a distinct organization at the plasma membrane of the two cell types, supporting the proposed role of the actin cytoskeleton in CHO cells but not in polarized MDCK cells.

### Calcium chelation affects apical GPI-AP organization

It has been shown that cholesterol-dependent Golgi clustering of GPI-APs is required for their apical sorting and for the subsequent organization at the apical surface (Paladino et al., 2014). More recently, we uncovered that calcium levels in the Golgi apparatus are crucial for GPI-AP Golgi clustering (Lebreton et al., 2021). Therefore, here, we asked whether calcium chelation alters apical GPI-AP organization in polarized MDCK cells. Strikingly, upon calcium chelation (60 min EGTA, 4 mM), we observed a significant decrease in the mean brightness (B) values of GFP-FR (from 1.29 to 1.13, *p* < 0.00001), which corresponds to a shift toward the monomeric/dimeric forms (Figure 3C), thus indicating disruption of homoclusters. Consistently, STORM experiments showed that PLAP became more randomly distributed upon calcium chelation (Figure 3D; Supplementary Figure S1D), indicating that they are not confined in defined areas anymore. We also found that under Ca^2+^ chelation, the hetero-FRET between GFP-FR and mCherry-PLAP was also lost (Figure 3E), indicating lack of heteroclusters in agreement with previous findings showing that homoclusters are required for heterocluster formation (Paladino et al., 2014). Thus, calcium depletion leads to a complete disruption of GPI-AP surface organization (homoclusters, heteroclusters, and area of confinement), which is restored after calcium replenishment (Figures 3C–E).

**FIGURE 4:**
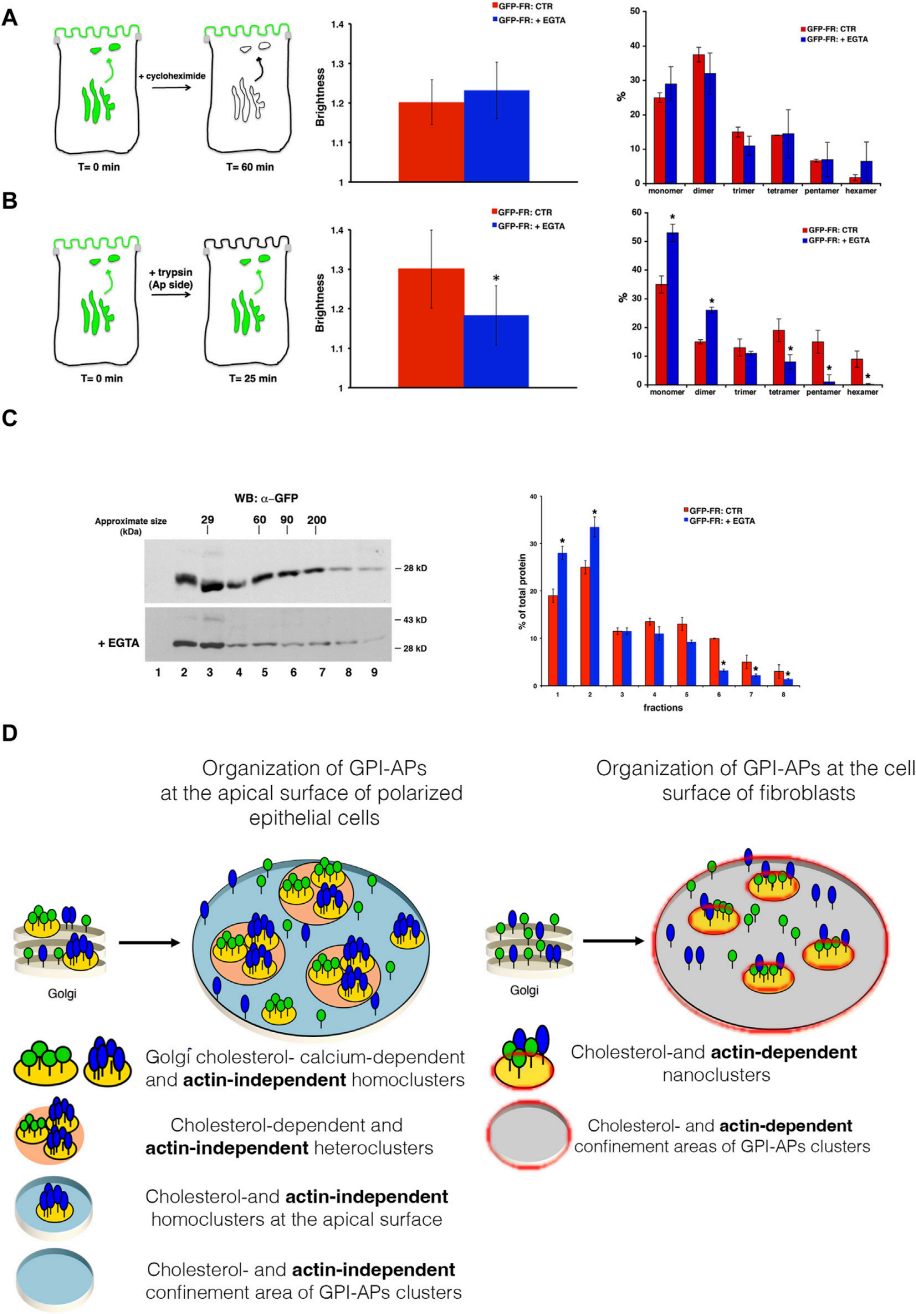
Calcium chelation impairs GFP-FR Golgi homoclusters in MDCK cells, underscoring different mechanisms of GPI-AP organization and regulations between epithelial and fibroblastic cells. N&B analysis of GFP-FR was performed on the plasma membrane **(A)** or in the Golgi **(B)** pool of the protein in polarized MDCK cells. **(A)** In order to consider exclusively the cell surface pool of GFP-FR, MDCK cells grown on filters were incubated with cycloheximide (90 min) before EGTA treatment (still in the presence of cycloheximide), as depicted in the scheme (on the left). In the middle, quantification of the brightness of GFP-FR from three independent experiments either in control conditions (red bar) or upon chelation of calcium (blue bar) is shown, *n* > 50 cells; error bars, ±SD. On the right, graphical representation of the percentage of pixels falling into different classes of B-values (from monomer to hexamer) on the basis of the calibration curve (Paladino et al., 2014). Values are expressed as the mean of three independent experiments, *n* > 30 cells; error bars, ±SD. **(B)** MDCK cells grown on filters were treated with trypsin 25 μg/mL for 25 min exclusively at the apical side in order to remove the pool of GFP-FR already present at the plasma membrane and to analyze the GFP-FR Golgi pool, as depicted in the scheme. Then, cells were untreated (CTR) or incubated with EGTA for 1h and imaged at the Golgi level. In the middle, quantification of the brightness of GFP-FR in the Golgi compartment from three independent experiments either in control conditions (CTR, red bar) or upon chelation of calcium (+EGTA, blue bar), *n* > 50 cells; error bars, ±SD. *p < 0.001, Student’s *t-*test. On the right, graphical representation of the percentage of pixels falling into different classes of B-values (from monomer to hexamer) on the basis of the calibration curve (Paladino et al., 2014). Values are expressed as the mean of three independent experiments; *n* > 30 cells. Error bars, ±SD. *p < 0.001, Student’s *t-*test. **(C)** MDCK cells grown on filters were treated as in **(B)** and purified on velocity gradient. Cells were lysed and run on velocity gradient, as described in methods. Fractions were collected from top (fraction 1) to bottom (fraction 9), TCA-precipitated, run on SDS-PAGE gel, and revealed by Western blotting with a specific antibody anti-GFP. Molecular weight markers are indicated on the top of the panels. The molecular weight of the monomeric form of GFP-FR is indicated together with the band at 43 kDa, which represents a partially denatured dimer of GFP. On the right panel, the distribution of GFP-FR in the fractions of the gradient is expressed as the percentage of total protein. Mean values of two independent experiments are shown. **(D)** Graphical representation of the mechanism of GPI-AP organization and regulation in polarized MDCK cells or in CHO cells.

At this point, we determined whether calcium chelation directly affects the organization of GPI-APs at the plasma membrane or whether the cluster dispersion at the plasma membrane resulted from the impairment of homoclustering in the Golgi apparatus. To discriminate between these two possibilities, we performed N&B analysis, considering only the plasma membrane pool of GFP-FR in cells pre-treated with cycloheximide to block new protein synthesis, as previously described (Paladino et al., 2014) (Figure 4A). In these conditions, the mean B-value of the plasma membrane pool of GFP-FR and the distribution between monomer and oligomer is comparable to that in the control condition and upon EGTA treatment (1.19 vs. 1.20) (Figure 4A), indicating that calcium chelation does not affect GFP-FR clusters at the plasma membrane. By performing N&B analysis at the level of the Golgi membranes where EGTA has been shown to induce mobilization of calcium (Chandra et al., 1991) see Materials and Methods), we could monitor a decrease in the B-value of GFP-FR with a shift toward monomers and dimers (mean B value = 1.20 vs. 1.06 control condition vs. EGTA treatment; *p* < 0.0001) (Figure 4B) consistent with a reduction in the Golgi GFP-FR high-molecular weight complexes purified by velocity gradient (Figure 4C). In summary, our data suggest that calcium chelation does not exert a direct impact on the plasma membrane clustering of GPI-APs. Instead, it influences the formation of Golgi GPI-AP homoclusters, subsequently impairing the organization of apical GPI-APs.

In conclusion, our comprehensive dataset reinforces the notion that discrete mechanisms govern the membrane organization of GPI-APs in epithelial and fibroblastic cells. Accordingly, we propose a model (depicted in Figure 4D) according to which in fibroblasts, actin- and cholesterol-driven GPI-AP nanoclusters organize directly at the cell surface. Conversely, in polarized MDCK cells, cholesterol- and calcium-dependent Golgi GPI-AP clusters regulate their organization at the apical surface independently of the actin cytoskeleton.

Finally, this different organization may be related to the way in which fibroblasts and epithelial cells respond to the plethora of external stimuli (e.g., chemical and mechanical) and adapt to various extracellular environments in order to perform their unique functions. The actin cytoskeleton plays a central role in determining and modulating these physiological responses, and its organization is extremely different in these cells, with prominent marginal circular bundles in epithelial cells compared to parallel bundles in fibroblasts, for example, Braga (2016) and Burridge (2017).

## Materials and methods

### Cell cultures, transfections, and antibodies

MDCK cells were grown in DMEM (Sigma-Aldrich) containing 5% FBS. MDCK cells were co-transfected with sequences encoding for GFP-FR and mCherry-PLAP or mCherry-p75 (Paladino et al., 2014). Differently transfected CHO cells, grown in HAM’s F12 medium 10% FBS, were used; cells stably expressing GFP-FR (kind gift of Dr. S. Mayor, NCBS Bangalore, India) were transiently co-transfected with mCherry-PLAP; CHO cells transiently transfected with c-DNA encoding for PLAP. We generated Fab fragments (using the protocol provided by Pierce) for PLAP antibody, and then, it was coupled to CY5 dye (GE Healthcare Life Science) and used in STORM experiments (Paladino et al., 2017).

### Perturbation of the actin cytoskeleton

To perturb the actin cytoskeleton, we incubated cells in culture medium at 37°C with the following compounds: 6 μM latrunculin A (molecular probes) for 5 min or 25 μM latrunculin A for 30 min (control cells were incubated in the culture medium containing equivalent concentrations of DMSO); 5 μM jasplakinolide (molecular probes) for 5 min; and 50 μM blebbistatin, a myosin II inhibitor, (Sigma) for 90 min.

### Velocity gradients

Velocity gradients were performed using a previously published protocol (Lebreton et al., 2008; Paladino et al., 2008). The cells were grown for 4 days in 100-mm dishes or on filters, washed in phosphate-buffered saline containing CaCl_2_ and MgCl_2_, and lysed on ice for 30 min in 20 m M Tris, pH 7.4, 100 m M NaCl, 0.4% SDS, and 0.2% Triton X-100. The lysates were scraped from dishes, sheared through a 26-gauge needle, and layered on top of a glycerol gradient (40%–20%) after removal of nuclei by low-speed centrifugation. After centrifugation at 45,000 rpm for 16 h in an ultracentrifuge (model SW 50; Beckman counter), fractions of 300 μL were harvested from the top of the gradient and trichloroacetic acid-precipitated. GFP-FR was revealed by Western blotting using the GFP antibody.

### Cholesterol measurement

We measured the amount of cellular cholesterol using a fluorescence- or a radioactive-based assay. For the fluorescence-based assay, polarized MDCK or CHO cells were incubated in the presence of TopFluor cholesterol (Avanti Polar Lipids) in delipidated cellular medium for 10 min prior to imaging. For the radioactive-based assay, cells stably expressing PLAP were incubated for 6 h with [^3^H]-cholesterol in the culture medium and then chased for 24 h in fresh culture medium. After lysis with buffer containing 1% TX-100, lysates were immunoprecipitated with a specific antibody against PLAP (from Rockland) in order to evaluate the amount of cholesterol in the PLAP surrounding. The radioactivity associated with PLAP immunoprecipitates and with total lysates was determined using a liquid scintillation counter.

### N&B experiments

The number and molecular brightness method, a technique based on moment analysis for the measurements of the average number of molecules and brightness in each pixel in fluorescence microscopy images (Digman et al., 2008), provides the state of aggregation of molecules in living cells with a high spatial and temporal resolution. N&B experiments were carried out as previously described (Paladino et al., 2014).

#### Microscopy and image analysis

In total, 50 frame time-series were acquired with a Zeiss LSM 510 META microscope equipped with a plan apo ×63 oil-immersion (NA 1.4) objective lens using the following settings: 488 nm Argon laser, 0.05 mW of output power, 505–550 nm emission, gain less or equal to 850, offset 0.1, and digital gain 1. Scanning parameters were as follows: 512 × 512 frame window, 25.61 μs/pixel dwell time, no average, zoom 6x, ROI (x, y) 256 × 64, and pinhole corresponding to 1-mm optical slice. Images were collected with resolutions of 70 nm/pixel. All measurements were performed in cells displaying comparable levels of fluorescence intensity. Data from each cell were analyzed using SimFCS software (Global Software, East Villa Grove, IL 61956, United States), following a described procedure (Digman et al., 2008). Correction was applied for taking into account the analog detection of fluorescence by the photomultiplier tubes of the confocal microscope in order to express the molecular brightness (e) in terms of photons/s/molecule (Dalal et al., 2008). In brief, the correction parameters S (the conversion factor between one photon detected and the number of digital levels produced by the electronics), offset, and sigma 0 were determined, for each experiment, plotting the measured average intensity (<I>) vs. average variance (<Var>) of 50 frame time-series acquired using the same settings as above except for four different values of laser transmission percentages and filters and beam splitters configured to get reflection images, in order to detect the defined amount of light originating directly from the laser. The obtained plots were linearly interpolated, and the equation of straight line (R ≥ 0.99) was used to extract the parameters S and offset based on the following equation: <Var> = S * <I> + q (parameter related to readout noise). The parameter sigma0 was estimated from time-series acquired with laser off as the half-maximum width of the histogram peak of the dark counts. Its value was constantly lower than 0.1 and consequently was approximated to zero in all the calculations.

In the analog system, brightness was calculated pixel by pixel from the following equation: B = V(x,y)/(S*Ix,y); the relationship with molecular brightness is described by the following equation: B/S = e+1. Here, we indicate the ratio B/S with the term brightness. Hence, the measured brightness (B/S) is > 1 from the pixels with mobile components, while B/S = 1 from the pixels with immobile features.

Photobleaching correction (photobleaching rate measured from the experimental data) has been included in the algorithms used to analyze N&B data (Digman et al., 2008). Specifically, we used a high-pass filter to the intensity as a function of time of each pixel, which we experimentally verified to be able to remove slowly varying signals. After removal of the trend, we added a constant equal to the average intensity at that pixel. Therefore, the variance of the “immobile” part is unaffected by bleaching after correction, and we can recover the variance of the mobile part (Digman et al., 2008).

In all experiments, a detrend function (the same used for bleaching correction) was applied to image stacks before determining the B-value in order to avoid that slow changes in the intensity due to the cell movement or protrusion/retraction events could interfere with our measurements (Dalal et al., 2008; Digman et al., 2008). Finally, all acquisitions where we monitored aberrant movements (e.g., microvilli movement or fluctuations of the apical membrane) were discarded.

#### Data analysis

As previously shown (Paladino et al., 2014) using MATLAB software (MathWorks Inc., Natick, MA) and the K-means function, we partitioned, with an interval of 0.5, the observed brightness values upon different experimental conditions into N exclusive groups with statistical reliability. In particular, for each experiment (number of cells > 15), we obtained the percentage of pixels in each group (calculated as the average of single-cell values from an experiment). The range of B-values was ascribed to monomer, dimer, and trimer on the basis of extrapolation of the standard curve obtained by plotting the experimental B-values for monomeric, dimeric, and trimeric GFP (mGFP, mGFP-mGFP, and mGFP-mGFP-mGFP) vs. number of units per aggregate [for detail see Supplementary Figure S4 in Paladino et al. (2014)].

### FLIM experiments

For FRET-FLIM, we used a multifocal multiphoton microscope combined with a time-gated detection, as previously described (Padilla-Parra et al., 2008; Paladino et al., 2014). In brief, the FRET-FLIM apparatus combines multifocal multiphoton excitation (TriM Scope, LaVision BioTec, Bielefeld, Germany) connected to an inverted microscope (IX 71, Olympus, Tokyo, Japan) and a fast-gated CCD camera (PicoStar, LaVision BioTec, Bielefeld, Germany). A mode-locked Ti:Sa laser at 80 Mhz frequency and at 950 nm for the excitation of GFP (Spectra Physics, France) was split into 2 to 64 beams using a 50/50 beam splitter and mirrors. A line of foci was then created at the focal plane, which can be scanned across the sample. A filter wheel of spectral filters (535AF45 for GFP) was used to select the fluorescence imaged onto a fast-gated light intensifier connected to a CCD camera (PicoStar, LaVision, Germany). All instrumentation was controlled by Imspector software developed by LaVision BioTec.

The gate of the intensifier (adjusted at 2 ns) was triggered by an electronic signal coming from the laser, and a programmable delay box was used to acquire a stack of five time-correlated images of the 10-ns fluorescence decay window for GFP lifetime and a stack of 10 time-correlated images of the 20-ns fluorescence decay window for TopFluor cholesterol (since the lifetime of this dye is approximately 4 ns). The acquisition time of the CCD camera was adjusted considering the fluorescence signal level. Regions of interest (ROIs) of 140 × 140 pixel (4-6 cells) were acquired.

Analysis of the data was done using ImageJ (Rasband, W.S., ImageJ, U. S. National Institutes of Health, Bethesda, Maryland, United States, http://rsb.info.nih.gov/ij/). The methodology used in order to perform quantitative analysis was previously developed by Padilla-Parra et al. (2008). In brief, the five or ten images coming from a time-gated stack are first smoothened by a 3 × 3 mask to decrease the noise and to recover mean lifetime, <t>; the following equation is applied:fd1

$$\langle \tau \rangle =\sum_{i=1}^{5}∆t_{i}I_{i}/\sum_{i=1}^{5}I_{i},$$

where $∆t_{i}=2i\;–\;1$ corresponds to the time delay after the laser pulse of the *i*th image acquired, N corresponds to the number of time-gated images (5 for GFP lifetime and 10 for TopFluor cholesterol lifetime), and I_i_ corresponds to the pixel intensity map in the *i*th image.

Both in N&B and FLIM experiments, MDCK and CHO cells, grown either on bottom-glass dishes or on filters, were imaged *in vivo* in CO_2_-independent medium (150 mM NaCl, 5 mM KCl, 1 mM, CaCl_2_, 1 mM MgCl_2_, and 20 mM HEPES, pH 7.4).

We always used confluent fully polarized MDCK cells grown either on plastic dishes or polycarbonate filters for 3–4 days, after which they attain complete polarization (Paladino et al., 2014). We focused on the apical membrane by finding first the focal plane corresponding to the filter (or to the opposite membrane plane which contains easily identifiable microvilli). Because CHO cells are very flat, we generally imaged these cells only at the cell periphery in order to detect only signals coming from the surface and to avoid intracellular contamination.

### STORM experiments

#### Microscopy system

STORM imaging was performed on a custom-built microscopy system featuring a Nikon Ti-E eclipse microscope body; 2 lasers at 647 nm (MPB Communications) and 405 nm (Oxxius), allowing sample illumination in wide-field; and an EMCCD camera (Andor iXon 897 ultra). The microscope is equipped with a perfect focus system (PFS, Nikon) to prevent the axial drift of the sample. Micromanager is used to control the microscope and the camera during image acquisition. The laser power is adjusted during image acquisition by controlling the AOTF (AA Optoelectronic) using a Python program (Lelek et al., 2012).

#### Sample preparation

STORM experiments were carried out as described in Paladino et al. (2017). Polarized MDCK or CHO cells were fixed with paraformaldehyde at 4% concentration in the presence of glutaraldehyde at 0.02% for 20 min and quenched with NH_4_Cl for 10 min, and after unspecific saturation (with gelatin at 0.2%), the cells were stained with Fab-GFP or Fab-PLAP coupled to the dye Cy5 for 45 min. Because the polarized cells are growing on a filter, specific mounting was needed for high-resolution microscopy. Multiwell slides with eight wells (MP Biomedicals, LLC) were used to keep the cells sufficiently close to the coverslip, considering the working distance of the objective lens. The filters were cut, and each piece matched the size of the hole engraved in the cover-glass. These holes were filled with an oxygen scavenger buffer, as previously described (van de Linde et al., 2011), which promotes fluorophore blinking when in contact with the filter and the cells. Fluorescent beads were added to the sample prior to mounting to allow estimation and computational correction of x-y drift *a posteriori*.

#### Imaging

Because the polarized cells were grown on the filter placed between coverslips, the apical surfaces of cells are not always close to the coverslip, making STORM imaging more difficult. To find polarized cells and avoid the axial drift during STORM acquisition, the coverslip is scanned along the *x* and *y*-axes with the PFS turned on until cells are found sufficiently close to the coverslip and in the offset range of the PFS. dSTORM imaging is performed as previously described (Lelek et al., 2012). Image acquisition parameters are as follows: EM camera gain 300; exposure time 100 ms; binning 1:1; in each experiment, a sequence of 30,000 to 50,000 full-sized raw images (512 × 512 pixels- 54 × 54 µms) was acquired. Raw images were processed to compute molecular localizations using PALMTT, a modified version of the MATLAB-based single-particle tracking software program MTT (Serge et al., 2008). Another in-house MATLAB program, PALMvis, was used to correct for sample drift and generate super-resolution visualizations.

### pc-STORM analysis

The computed localization data were further subjected to a pair correlation analysis by adapting the procedure described in Sengupta et al. (2011). Normalized pair-wise correlation functions *g(r)* were computed from the localization data and then fitted to each of the following two equations:

$$g\left(r\right)=\frac{1}{4.\pi .\sigma_{s}^{2}.\rho }.\exp\left(\frac{-r^{2}}{4.\sigma_{s}^{2}}\right)+1$$ (1)

and

$$g\left(r\right)=\frac{1}{4.\pi .\sigma_{s}^{2}.\rho }.\exp\left(\frac{-r^{2}}{4.\sigma_{s}^{2}}\right)+\left(A.\exp\left(\frac{-r}{\xi }\right)+1\right)⊛\frac{1}{4.\pi .\sigma_{s}^{2}}.\exp\left(\frac{-r^{2}}{4.\sigma_{s}^{2}}\right).$$ (2)

Equation 1 is the correlation function expected for a random distribution of isolated (non-clustered) molecules, where *σ*_*s*_ is the standard deviation of random localization errors and *ρ* measures the density (number of localizations per unit surface). Eq. 2 is the correlation function expected for clustered molecules, where ξ measures the cluster size, and A is the amplitude of the protein correlation extrapolated to distance r = 0. Fitting Eq. 1 to pair correlation functions obtained for isolated molecules yields an estimate of *σ*_*s*_ (along with *ρ*), which is then held constant when fitting Eq. 2. The latter fitting, thus, yields three parameters: *A*, *ρ*, and *ξ*. It should be noted that in contrast to the original method (Sengupta et al., 2011), the experimental pc-STORM curves are normalized.

Squared regions of 4 μm × 4 μm on the cell membrane were selected manually based on the STORM localization image (e.g., Figure 2A upper panels on the left). For each area, we computed the pair-wise correlation function of these localizations and then fitted the random and clustered models to these curves (e.g., Figure 2A lower panels on the left). For each pair-correlation curve, the mean squared error is computed between the data and each of the two fitted models. The distribution of errors for the random and the clustered model is each represented by a histogram (e.g., Figure 2A upper panels on the right). The two distributions are then compared using a Kolmogorov–Smirnov test in order to determine if the clustered model fits the data significantly better than the random model. If this difference is not significant, we categorize the molecular distributions as random. Otherwise, we categorize it as clustered and use the fitted parameters ξ to determine the cluster sizes.

### Statistical analysis

In N&B and FRET-FLIM experiments, we used the two-tailed student test as statistical analysis. In pc-STORM analyses, Kolmogorov–Smirnov tests are used to determine if the data are better fit by a clustered than a random model (see above), and Wilcoxon tests are computed to compare distributions of cluster sizes between different conditions.

## Data Availability

The original contributions presented in the study are included in the article/Supplementary Material; further inquiries can be directed to the corresponding authors.

## Supplementary material

SUPPLEMENTARY FIGURE S1 Severe actin perturbation does not alter GPI-AP clustering in polarized MDCK cells. MDCK GFP-FR grown on filters were treated or not with latrunculin A 25 μM for 30 minutes, and then, the cells were either stained for actin cytoskeleton **(A)** using rhodamine phalloidin (as in Figure 1B) or lysed and run on velocity gradient as described in methods **(B)**. Fractions were collected from the top (fraction 1) to the bottom (fraction 9), TCA-precipitated, run on SDS-PAGE gel, and revealed by Western blotting with a specific antibody anti-GFP. Molecular weight markers are indicated together with the band of 43 kDa, which represents a partially denatured dimer of GFP. Below, the distribution of GFP-FR in the fractions of the gradient is expressed as the percentage of total protein. Mean values of two independent experiments are shown. Error bars, ±SD. NS, not significant. **(C)** Violin plots show the distribution of 2.354**σs* obtained by fitting the random model, where *σs* is the precision of localization and 2.354 the theoretical constant of a Gaussian distribution. Distribution of 2.354*σ*s* obtained by fitting the random model to all 4 µm× 4 µm regions in MDCK cells expressing p75-NTR (as monomer reference molecule) or in CHO cells expressing PLAP upon latrunculin exhibits a similar box plot. **(D)** Violin plots resulting from the fit of the random model to all 4 µm× 4-µm regions in MDCK cells expressing p75-NTR (used as monomer reference molecule, Paladino et al. 2017) or expressing PLAP upon calcium chelation are similar, revealing the random organization of PLAP upon EGTA treatment.
