# Supplementary figures and images for: Actin cytoskeleton differently regulates cell surface organization of GPI-anchored proteins in polarized epithelial cells and fibroblasts
Source: Front Mol Biosci. 2024 May 7;11:1360142. doi: 10.3389/fmolb.2024.1360142 (PMC11106487; doi:10.3389/fmolb.2024.1360142)

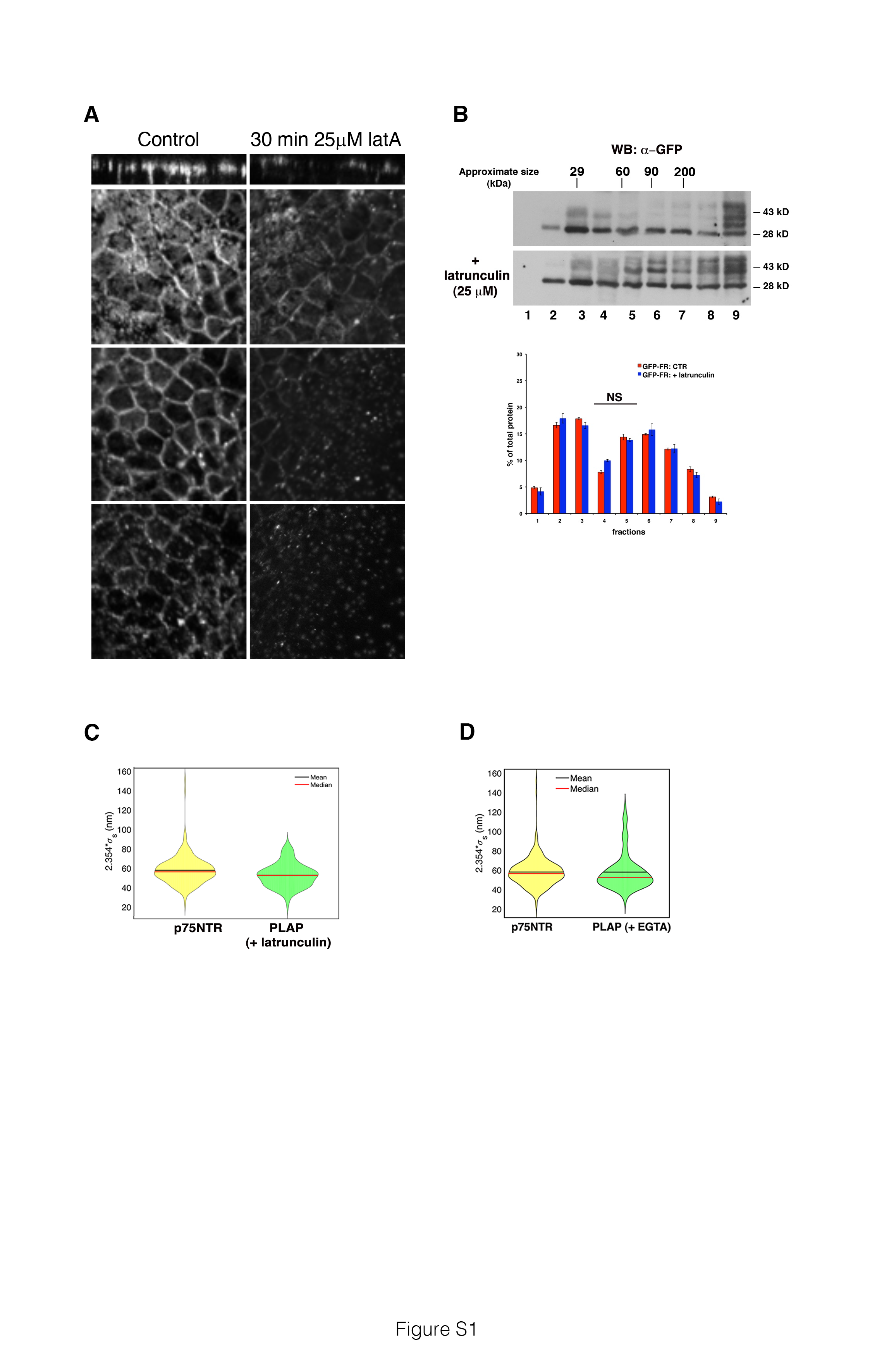

Supplement: Supplementary file 1 [file Image1.TIF]
